# Insect Cells for High-Yield Production of SARS-CoV-2 Spike Protein: Building a Virosome-Based COVID-19 Vaccine Candidate

**DOI:** 10.3390/pharmaceutics14040854

**Published:** 2022-04-13

**Authors:** Bárbara Fernandes, Rute Castro, Farien Bhoelan, Denzel Bemelman, Ricardo A. Gomes, Júlia Costa, Patrícia Gomes-Alves, Toon Stegmann, Mario Amacker, Paula M. Alves, Sylvain Fleury, António Roldão

**Affiliations:** 1 iBET, Instituto de Biologia Experimental e Tecnológica, Apartado 12, 2780-901 Oeiras, Portugal; bfernandes@ibet.pt (B.F.); rcastro@ibet.pt (R.C.); ragomes@ibet.pt (R.A.G.); palves@ibet.pt (P.G.-A.); marques@ibet.pt (P.M.A.); 2 ITQB NOVA, Instituto de Tecnologia Química e Biológica António Xavier, Universidade Nova de Lisboa, Av. da República, 2780-157 Oeiras, Portugal; jcosta@itqb.unl.pt; 3 Mymetics BV, J.H. Oortweg 21, 2333 CH Leiden, The Netherlands; farien.bhoelan@mymetics.com (F.B.); denzel.bemelman@mymetics.com (D.B.); toon.stegmann@mymetics.com (T.S.); 4 Mymetics SA, Route de la Corniche 4, 1066 Epalinges, Switzerland; mario.amacker@mymetics.com (M.A.); sylvain.fleury@mymetics.com (S.F.); 5 Department of Biomedical Research (DBMR), Department of Pulmonary Medicine, Bern University Hospital, University of Bern, 3008 Bern, Switzerland

**Keywords:** IC-BEVS, spike protein, virosomes, protein production

## Abstract

The severe acute respiratory syndrome coronavirus 2 (SARS-CoV-2) homotrimeric spike (S) protein is responsible for mediating host cell entry by binding to the angiotensin-converting enzyme 2 (ACE2) receptor, thus being a key viral antigen to target in a coronavirus disease 19 (COVID-19) vaccine. Despite the availability of COVID-19 vaccines, low vaccine coverage as well as unvaccinated and immune compromised subjects are contributing to the emergence of SARS-CoV-2 variants of concern. Therefore, continued development of novel and/or updated vaccines is essential for protecting against such new variants. In this study, we developed a scalable bioprocess using the insect cells-baculovirus expression vector system (IC-BEVS) to produce high-quality S protein, stabilized in its pre-fusion conformation, for inclusion in a virosome-based COVID-19 vaccine candidate. By exploring different bioprocess engineering strategies (i.e., signal peptides, baculovirus transfer vectors, cell lines, infection strategies and formulation buffers), we were able to obtain ~4 mg/L of purified S protein, which, to the best of our knowledge, is the highest value achieved to date using insect cells. In addition, the insect cell-derived S protein exhibited glycan processing similar to mammalian cells and mid-term stability upon storage (up to 90 days at −80 and 4 °C or after 5 freeze-thaw cycles). Noteworthy, antigenicity of S protein, either as single antigen or displayed on the surface of virosomes, was confirmed by ELISA, with binding of ACE2 receptor, pan-SARS antibody CR3022 and neutralizing antibodies to the various epitope clusters on the S protein. Binding capacity was also maintained on virosomes-S stored at 4 °C for 1 month. This work demonstrates the potential of using IC-BEVS to produce the highly glycosylated and complex S protein, without compromising its integrity and antigenicity, to be included in a virosome-based COVID-19 vaccine candidate.

## 1. Introduction

Severe acute respiratory syndrome coronavirus 2 (SARS-CoV-2) is a highly pathogenic virus responsible for the coronavirus disease 2019 (COVID-19). In March 2020, COVID-19 was declared a pandemic by the World Health Organization (WHO) and, as of March 2022, more than 400 million persons were infected worldwide and 6 million people have died [1,2]. Although vaccines against COVID-19 have been already approved for human use [3], the emergence of new SARS-CoV-2 variants demands constant update or development of novel vaccine formulations.

Antigens displayed on particles, especially if present as repeat arrays, elicit stronger immune responses when compared to free soluble antigens, thus being a potentially complementary approach to the recently approved protein-based Novavax vaccine [4,5]. This repetitive unit display was successfully applied in licensed virosomal vaccines with an effective and long-lasting immunogenicity and an excellent safety profile [6,7]. In this particular case, virosomes were lipid-based particles derived from reconstituted influenza virus membranes with a diameter of about 100 nm, which acted as carrier to a variety of antigens. This vaccine modality combines the advantages of a virus-like particle with the benefits of in vitro assembly from purified components, allowing to present antigens and adjuvants on the same particle while minimizing unspecific immune activation [8,9,10,11].

The SARS-CoV-2 homotrimeric spike (S) protein is the key antigen to target in a COVID-19 vaccine [12] since it mediates host cell infection by binding to the angiotensin-converting enzyme 2 (ACE2) [13,14]. S protein is highly glycosylated, with its glycans playing an important role in protein folding, stability, immune recognition and evasion due to epitope shielding [15,16,17].

Developing a virosomal-based vaccine against COVID-19 requires the production of significant amounts of high-quality and stable S protein, thus encouraging the use of high-yield expression systems and/or the design of new, improved production and purification strategies to meet such demand.

Different expression systems have been reported for full-length S protein production, the majority being mammalian cell-based [18]. The insect cell-baculovirus expression vector system (IC-BEVS) can be a valid alternative as it is commonly regarded as a low-cost and scalable production platform with an already proven track record [19,20]. Proteins expressed in insect cells can undergo post-translational modifications such as protein N-glycosylation; however, they present smaller and less processed glycans, designated as paucimannose glycans, respective to mammalian cells [21]. The production yields achieved (<1.5 mg/L of culture) in IC-BEVS are still below the desirable levels for the development and manufacturing of vaccines at commercial-scale [12,22]. In addition, different studies have described difficulties in obtaining stable, soluble S protein trimers due to their tendency to disassemble upon storage [18].

In this work, aiming to develop a scalable bioprocess to produce high-quality S protein for inclusion in a virosome-based COVID-19 vaccine candidate, different signal peptides, baculovirus transfer vectors, cell lines, infection strategies and formulation buffers were explored. An in-depth characterization of the produced protein was performed to assess its stability, oligomeric state and binding capacity to ACE2 receptor and selected neutralizing SARS-CoV-2 antibodies. In addition, the S protein was covalently coupled via its His-tag to a click chemistry lipid present in the virosomal membrane. The particle binding capacity to selected neutralizing SARS-CoV-2 antibodies was assessed.

## 2. Materials and Methods

### 2.1. Cell Lines and Culture Media

Insect *Sf*-9 (Invitrogen, Waltham, MA, USA), ExpiSf9™ (Thermo Fisher Scientific, Waltham, MA, USA) and SuperSf9-2 (Oxford Expression Technologies, Oxford, UK) cells were routinely sub-cultured at 0.4–1 × 10^6^ cell/mL every 3–4 days when cell density reached 2–5 × 10^6^ cell/mL in 500 mL shake flasks (10% working volume, *w/v*) in a Innova 44R incubator (orbital motion diameter of 2.54 cm- Eppendorf) at 27 °C and 100 rpm. Sf-900^TM^ II SFM (Thermo Fisher Scientific, Waltham, MA, USA) and ExpiSf ^TM^ CD (Thermo Fisher Scientific, Waltham, MA, USA) media were used to culture *Sf*-9 and SuperSf9-2 cells, and ExpiSf9™ cells, respectively.

### 2.2. Recombinant Baculovirus

#### 2.2.1. Expression Vectors

SARS-CoV-2 spike (S) protein sequence (GenBank MN 908947) is described in Supplementary Information: The S protein sequence was modified by eliminating the furin cleavage site between S1 and S2, introducing the mutations K968P and V987P, truncation of the protein after Q1208 and introduction of the HIV gp160 derived fusion clamp (Watterson et al., 2021) after Q1028, followed by the 6 histidine tag (6his-tag), for an S protein with an expected MW of 140 kD. Three signal peptides were tested: (1) honeybee melittin (BVM) insect-derived; (2) gp67 of *Autographa californica* nuclear polyhedrosis virus (AcMNPV); and (3) native S signal peptide. Three baculovirus transfer vectors were tested: (1) pOET1, the S protein gene is under the control of AcMNPV polyhedrin (polh) promoter; (2) pOET3, the S protein gene is under the control of AcMNPV basic (p6.9) promoter; and (3) pOET5, one copy of the S protein gene is under the control of AcMNPV polh promoter and another copy is under the control of p10 promoter. The five different expression plasmids evaluated in this study, synthetized by GenScript, are described in Table 1.

#### 2.2.2. Baculovirus Generation

Recombinant baculoviruses (rBac) were generated using the flashback ULTRA^TM^ system (Oxford Expression Technologies, Oxford, UK) according to the manufacturer’s instruction. Amplification of baculovirus stocks was performed as described elsewhere [23]. Briefly, *Sf*-9 cells were infected at cell concentration of 1 × 10^6^ cells/mL and at a multiplicity of infection (MOI) of 0.01–0.1 pfu/cell. When cell viability reached 80–85%, cultures were harvested, centrifuged at 200× *g* for 10 min at 4 °C. The pellet was discarded, and the supernatant was centrifuged at 2000× *g* for 20 min at 4 °C. The resulting supernatant was stored at 4 °C until further use.

### 2.3. Protein Production and Purification

S protein production using *Sf*-9, ExpiSf9™ and SuperSf9-2 was performed in shake flasks (SF; 500 mL, 10% *w/v*) and in 20 L stirred tank bioreactors (STB). Cells were seeded at 0.3–0.6 × 10^6^ cells/mL and infected with rBac at different cell concentrations at the time of infection (CCI, 1 × 10^6^ and 2 × 10^6^), and MOI (0.1 and 1 pfu/cell).

Bioreactor cultures were performed in a computer-controlled BIOSTAT^®^ Cplus 20 L vessel (Sartorius, Göttingen, Germany) equipped with two Rushton impeller and a ring-sparger for gas supply. The pH was monitored (not controlled) along culture time. The partial pressure of oxygen (pO_2_) was set to 30% of air saturation and was maintained by varying the agitation rate (70 to 250 rpm), the percentage of O_2_ in the gas mixture (0 to 100%) and the gas flow rate was set to 0.01 vessel volumes per minute (vvm). The temperature was kept at 27 °C and the working volume was 20 L. Cell concentration and viability were measured daily, and culture samples processed and stored as described elsewhere [24]. The bioreactor culture was harvested 2–3 days post-infection when cell viabilities reached 70%.

Purification of secreted S protein was carried out on ÄKTA systems (Cytiva, Marlborough, MA, USA) as described elsewhere (Castro et al., 2021). Cell culture bulk was harvested by filtering through 0.45 and 0.22 μm Sartopore 2 (Sartorius, Göttingen, Germany). Tangential flow filtration (TFF) was used to concentrate and dialyze the clarified supernatants to 50 mM sodium phosphate supplemented with 500 mM NaCl and 20 mM imidazole, at pH 7.4 (binding buffer). S protein was purified by immobilized metal ion affinity chromatography on a Histrap HP or Nickel Sepharose HP column (5 mL volume; Cytiva, Marlborough, MA, USA) equilibrated with binding buffer. Two washing steps with 35 and 50 mM imidazole were performed, and S protein was eluted with a linear gradient up to 500 mM imidazole. The eluate was concentrated using a Vivaflow^®^200 of 50 kDa crossflow device (Sartorius, Göttingen, Germany), incubated 30 min at 4 °C with 5 mM EDTA and purified by size exclusion chromatography (SEC) using Superdex 200 (GE Healthcare, Chicago, IL, USA) previously equilibrated with 10 mM HEPES, 150 mM NaCl, at pH 7.2. The eluate was concentrated using Vivaflow^®^200 of 50 kDa crossflow device and filtered through a polyethersulfone membrane with 0.2 μm. Purified S protein was formulated in 10 mM HEPES, 150 mM NaCl at pH 7.2 with 10% glycerol, and stored at −80 °C until further analysis.

### 2.4. Virosomes Preparation

Virosomes were prepared as described earlier [25]. Briefly, inactivated influenza virus A/Brisbane/59/2007 (Seqirus, Parkville, Australia) was solubilized with the detergent octaethyleneglycol-mono(n-dodecyl)ether (OEG) and the viral nucleocapsid was removed by centrifugation. To the supernatant, the lipid 1,2-dioleoyl-sn-glycero-3-phosphocholine (DOPC) (Merck) and the click chemistry lipid dicyclobenzooctyl-phosphatidylethanolamine (DBCO-PE) (Avanti Polar Lipids, Alabaster, AL, USA) were dissolved in OEG, and added OEG was then removed by batch chromatography on polystyrene beads (BioBeads SM2) as described; resulting homogenous virosomes were sterilized by 0.22 µm filtration.

2-azidoethyl thiophosphodichlorate (ATPD) was synthesized and purified as described (Jia, S. et al., 2020) by Acme Bioscience (Palo Alto, CA, USA). S protein was dialyzed against 50 mM HEPES pH 8.5 for 4 h and then mixed with ATPD at a 200:1 ratio of ATPD to protein for 1 h at RT. The product was dialyzed overnight against 2000 volumes of buffer (145 mM NaCl, 5 mM HEPES, 1 mM EDTA, pH 7.4). The resulting S-azide conjugate product was incubated with virosomes for at least 24 h at 25 °C resulting in covalent coupling of S to virosomes through azide-DBCO-PE click chemistry.

### 2.5. Analytics

#### 2.5.1. Cell Concentration and Viability

Cell concentration and viability were analyzed by trypan blue dye exclusion method [26] using a Cedex HiRes Analyzer (Roche, Basel, Switzerland).

#### 2.5.2. Baculovirus Titration

Baculovirus titers were determined using the MTT assay [27,28].

#### 2.5.3. Protein Concentration

The concentration of purified S protein was determined by spectrophotometry at 280 nm on the mySPEC (VWR).

#### 2.5.4. Western Blot

Culture samples were centrifuged at 200× *g* for 10 min and supernatants collected and stored at −80 °C until further analysis. Western blot analysis was performed as reported elsewhere [29]. For S identification, the human monoclonal antibody SARS-CoV-2 spike protein (MA5-35948, Thermo Scientific, Breda, The Netherlands) was used at dilution of 1:3000. For 6his-tag recognition on S proteins identification, a mouse monoclonal antibody anti-6His tag (MA1-21315, Thermo Scientific, Breda, The Netherlands) was used at a dilution of 1:1000. As secondary antibody, an anti-mouse IgG (A3438, Sigma, Amsterdam, The Netherlands) and an anti-human IgG antibody (A9544, Sigma, Amsterdam, The Netherlands) conjugated with alkaline phosphatase were used at a dilution of 1:5000.

#### 2.5.5. Differential Scanning Fluorimetry

Differential scanning fluorimetry (DSF) was performed in a QuantStudio 7 Flex Real-Time PCR System (Thermo Fisher Scientific, Waltham, MA, USA), with excitation and emission wavelengths of 580 and 623 nm, respectively, using a MicroAmp^TM^ EnduraPlate^TM^ Optical 96-Well Fast Clear Reaction Plate with Barcode (Thermo Fisher Scientific, Waltham, MA, USA). The samples were heated from 25 °C to 90 °C with stepwise increments of 0.016 °C per second, followed by the fluorescence read out. For each well, 20 µL final volume with 4 µg of S protein and 4-fold of ROX™ Passive Reference Dye (Thermo Fisher Scientific, Waltham, MA, USA) was prepared with protein purification buffer. The assays were carried out in triplicates and the results were analyzed in the Protein Thermal Shift^TM^ Software V1.3.

#### 2.5.6. HPLC-SEC

Purified S protein was analyzed using a HPLC system equipped with Photodiode Array Detector (Waters, Milford, MA, USA). S protein samples were injected in a XBridge BEH 450 Å SEC 3.5 µm HPLC column (Waters) equilibrated in 10 mM HEPES with 150 mM NaCl at pH 7.2. The system flow rate was maintained at 0.86 mL/min and eluted proteins were detected at 280 nm. Twenty micrograms of protein was injected in each HPLC run.

#### 2.5.7. Site-Specific Glycosylation Analysis

Purified S protein was analyzed by LC-MS as described in [30]. For glycans identification, the N-glycans database described in CFG Functional Glycomics Gateway (http://www.functionalglycomics.org/fg/, accessed on 2 October 2021) with Spodoptera taxonomic restriction was used. MS data were analyzed using the BioPharmaView software (BPV, Version 3.0, SCIEX) and the protein sequence of spike. Glycans were identified using MS1 data (considering a peptide deconvolution tolerance of 10 ppm, XIC *m/z* width of 0.025 Da and *m/z* tolerance of 5 ppm) and fragmentation MSMS data (considering a MSMS tolerance of 0.03 Da). All MSMS data were manually examined for the presence of MSMS specific glycan marker ions. The data was also manually examined for consistence in retention time information and spectrum quality.

#### 2.5.8. ELISA

For the epitope mapping ELISA on S protein, ELISA plates (Nunc high-binding, Thermo-Fisher) were coated overnight with purified S protein at 0.5 µg/mL in phosphate buffered saline (PBS). The plates were then washed with PBS containing 0.05% Tween-20 (PBST, Sigma, Amsterdam, The Netherlands) and blocked with 5% Protifar (Nutrica, Utrecht, The Netherlands) in PBST for 2 h at RT. After washing in PBST, human monoclonal antibodies and ACE-2-Fc chimeric protein were added for one hour at RT, and revealed with goat anti-human antibodies coupled to horse radish peroxidase (HRP).

Coupling of the S protein to the virosomes was assessed by an ELISA in which the mouse monoclonal antibody 395-F2-04/03 (CePower) toward the hemagglutinin on virosomes was used to coat ELISA plates for capturing virosomes. The plates were then washed with PBST and blocked with 5% Protifar (Nutrica, Utrecht, The Netherlands) in PBST, then incubated with virosomes for 1 h at RT and further processed as described above. During the ELISA, the virosomes remained intact.

Antibodies tested were: ACE2-NN-IgGFc (Absolute Antibody, Redcar, UK), CR3022 (Abcam, Cambridge, UK), all SARS-CoV-2 antibodies (courtesy of prof. Sanders, Academic Medical Center, Amsterdam, The Netherlands) [31].

### 2.6. Statistical Analysis

Data were expressed as mean ± standard deviation. Differences were tested by One-Way ANOVA with post hoc Tukey’s multiple comparison analysis method and Dunnett’s multiple comparison test (adjusted *p*-value < 0.05 was considered statistically significant) or by t-test unpaired assuming Gaussian distribution (adjusted *p*-value < 0.05 was considered statistically significant).

## 3. Results

### 3.1. SARS-CoV-2 Spike Protein Production in Insect Cells

Aiming to improve S protein production yields in insect cells, different signal peptides, baculovirus transfer vectors, cell lines, infection strategies and formulation buffers were evaluated.

#### 3.1.1. Infection Strategy

**Figure 1:**
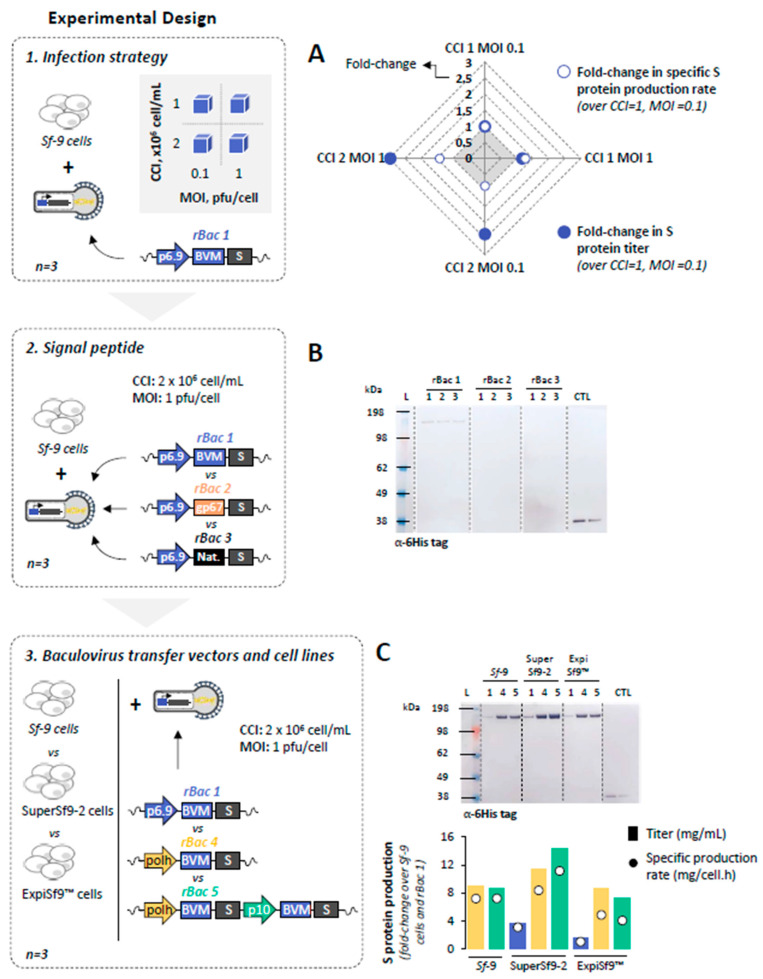
Production of SARS-CoV-2 spike (S) protein in small-scale shake flasks. (**A**) S protein titers and specific production rates at different cell concentrations at infection (CCIs) and multiplicity of infections (MOIs). (**B**) Identification of S protein in culture supernatant samples collected at time of harvest by Western blot; numbers 1–3 denote culture replicates. (**C**) Identification of S protein in culture supernatant samples collected at time of harvest by Western blot; numbers 1, 4 and 5 denote rBAC used (**upper panel**), and S protein titers (bars) and specific production rates (circles) using different cell lines and baculoviruses (**lower panel**). Color code: blue, yellow and green represents data using rBAC 1, 4 and 5, respectively. For Western blot analysis, a mouse monoclonal 6-Histag antibody was used; positive control (CTL) is an in-house purified protein with a hexahistidine tag in the C-terminal at 0.2 and 0.1 µg; Ladder (L) is SeeBlue™ Plus2 Pre-stained Protein Standard. The expected MW of S protein monomer is approximately 140 kDa. Data are expressed as mean ± standard deviation (relative to three biological replicates, *n* = 3).

To identify the best infection strategy, *Sf*-9 cells were infected at cell concentrations at infection (CCI) of 1 and 2 × 10^6^ cell/mL with recombinant baculovirus rBac 1 (Table 1) using multiplicities of infection (MOI) of 0.1 and 1 pfu/cell, and their growth and S protein expression kinetics assessed in small-scale shake flasks (SF) (Figure 1). Traditional profiles of insect cell growth and viability upon infection were observed, with S protein being identified by Western blot only in experiments at CCI of 2 × 10^6^ cell/mL (Supplementary Figure S1A). Maximum S protein titers and specific production rates were achieved for CCI = 2 × 10^6^ cell/mL and MOI = 1 pfu/cell (Figure 1A) and, therefore, this infection strategy was used in subsequent studies.

#### 3.1.2. Signal Peptide

Three different signal peptides were explored: the insect BVM (rBac 1), the rBac gp67 (rBac 2), and the native SARS-CoV-2 S protein signal peptide (rBAC 3) (Table 1). Insect *Sf*-9 cells were infected at CCI = 2 × 10^6^ cell/mL with each rBac at MOI = 1 pfu/cell, and their growth and S protein expression kinetics assessed in small-scale SF (Figure 1). Traditional profiles of insect cell growth and viability upon infection were observed (Supplementary Figure S1B), with S protein being only identified by Western blot in samples following infection with rBac 1 (Figure 1B); thus, baculovirus constructs using the BVM signal sequence were used for subsequent experiments.

#### 3.1.3. Baculovirus Transfer Vectors and Cell Lines

Three baculovirus transfer vectors, i.e., pOET1, pOET3 and pOET5 (Table 1), and three insect cell lines, i.e., *Sf*-9, SuperSf9-2 and ExpiSf9™, were evaluated for S protein production in small-scale SF using CCI = 2 × 10^6^ cell/mL and MOI = 1 pfu/cell (Figure 1). While baculovirus transfer vectors seem to have negligible impact on cell growth kinetics, the same does not withstand for the cell lines tested (Supplementary Figure S1C). Despite these differences, the S protein could be identified by Western blot in all experiments performed. Noteworthy, maximum S protein titer and specific production rate was achieved using SuperSf9-2 cells and rBac 5, where the S protein gene is duplicated and under the AcMNPV very late promoter polyhedrin (polh) and the p10 (Figure 1C).

#### 3.1.4. Formulation Buffer

**Figure 2:**
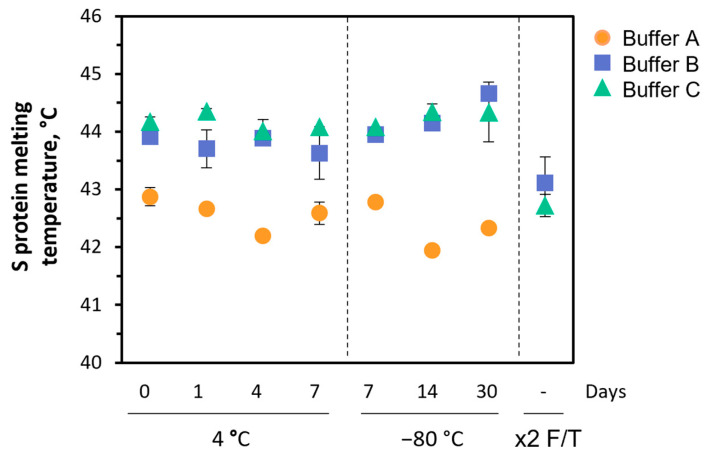
SARS-CoV-2 spike (S) protein thermal stability. Differential scanning fluorimetry analysis of S protein formulated in three different buffers: Buffer A: 10 mM HEPES + 150 mM NaCl at pH 7.2 (orange); Buffer B: 10 mM HEPES + 150 mM NaCl at pH 7.2, 10% glycerol (blue); and Buffer C: 10 mM HEPES + 150 mM NaCl at pH 7.2, 10% sucrose (green). Data are expressed as mean ± standard deviation (relative to three replicates measurements, *n* = 3).

Three formulation buffers were evaluated for S protein storage (Table 1): Buffer (A) 10 mM HEPES + 150 mM NaCl at pH 7.2; Buffer (B) 10 mM HEPES + 150 mM NaCl at pH 7.2, 10% glycerol; and Buffer (C) 10 mM HEPES + 150 mM NaCl at pH 7.2, 10% sucrose. S protein thermal denaturation was analyzed by DSF, with Buffers B and C showing consistently higher melting temperatures than Buffer A irrespective of storage time (up to 30 days) and temperature (−80 and 4 °C) (Figure 2). Noteworthy, S protein stored in Buffer A showed extensive degradation after two freeze-thaw cycles, being impossible to estimate its melting temperature, contrasting with S protein stored in Buffers B and C that only showed a 1 °C reduction in its melting temperature. For being slightly better, Buffer B was selected as formulation buffer for subsequent experiments.

### 3.2. Scale-Up SARS-CoV-2 Spike Protein Production

The feasibility of producing S protein in SuperSf9-2 cells was demonstrated in controlled, scalable 20 L stirred-tank bioreactors (STB); small-scale SF were used as control.

**Figure 3:**
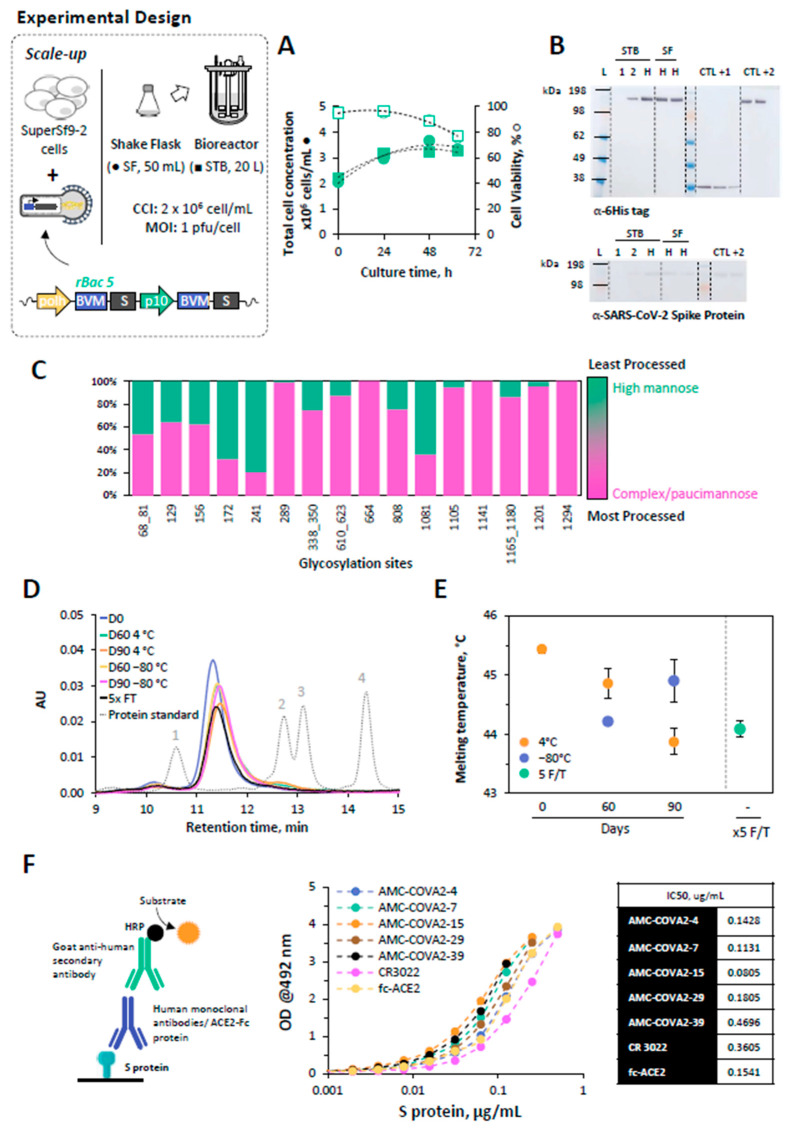
Production of SARS-CoV-2 spike (S) protein in a 20 L stirred-tank bioreactor (STB). (**A**) Cell growth kinetics upon infection. (**B**) Identification of S protein in supernatant samples collected at day 1, 2 and at time of harvest (H, 63 hpi) from STB and shake-flask (SF) cultures by Western blot; a mouse monoclonal 6-Histag antibody and a human monoclonal SARS-CoV-2 S antibody were used; positive controls were an in-house purified protein with a hexahistidine tag in the C-terminal at 0.2, 0.1 and 0.05 µg (CTL + 1) and an in-house purified spike protein with a hexahistidine tag in the C-terminal at 1 and 0.5 ng (CTL + 2); Ladder (L) is SeeBlue™ Plus2 Pre-stained Protein Standard; the expected MW of S protein monomer is approximately 140 kDa; STB and SF denote stirred-tank bioreactor. SF denote samples from shake-flask control 1 and 2, respectively. (**C**) Site-specific glycan analysis of S protein by mass spectrometry; glycans were grouped in categories: high mannose glycan series—M9 to M5; Man_9_GlcNAc_2_ to Man_5_GlcNAc_2_ (green), and complex/paucimannose glycans (pink). (**D**) HPLC-SEC analysis of S protein upon storage at different temperatures and upon ×5 freeze-thaw (T/T) cycles; dashed grey line represents the retention time of a protein standard mix: (1) thyroglobulin (660 kDa), (2) uracil (112 kD), (3) ovalbumin (44.2 kDa) and (4) ribonuclease A (13.7 kDa). (**E**) S protein thermal stability using differential scanning fluorimetry; data are expressed as mean ± standard deviation (relative to three replicates measurements, *n* = 3). (**F**) Binding of non-overlapping human neutralizing antibodies recognizing epitopes in the receptor binding domain of S protein (i.e., ACE2-NN-IgGFc, CR3022, all SARS-CoV-2 antibodies) or an ACE2-Fc chimeric protein binding to S protein bound to ELISA plates, and developed with goat anti-human HRP.

Cell growth and viability kinetics were comparable in both culture systems (Figure 3A). In addition, S protein could be identified by Western blot in both STB and SF cultures, with apparent similar band intensities (Figure 3B).

S protein produced in the 20 L STB was purified and a final production yield of 4.1 mg/L could be achieved, with removal of >95% of infectious particles, total deoxyribonucleic acid (DNA) and baculovirus genome copies. Purified S protein showed purity above 95% and a molecular weight of 383 kDa suggesting that S protein is in trimer conformation.

#### 3.2.1. Glycosylation Pattern of S Protein

Purified S protein was characterized by LC-MS for determination of site-specific glycosylation and glycan composition for all N-linked glycan sites previously described in the literature [15,17,30]. The glycosylation sites of S protein and their main glycan processing, subdivided into high mannose and complex/paucimannose-type glycosylation, are presented in Figure 3C. 21 N-glycosylation sites were found occupied and the detailed glycan compositions are described in Supplementary Table S1. A mixture of high mannose- and complex/paucimannose-type glycans was found at glycosylation sites N 68_81, N172, N241, N1081; the remaining 15 sites were dominated by processed, complex-type glycans.

#### 3.2.2. Mid-Term Storage Stability of S Protein

Mid-term storage stability of purified S protein was assessed by HPLC-SEC and DSF. HPLC-SEC analysis revealed a single peak in all conditions tested, thus, suggesting that S protein trimer conformation is maintained up to 90 days when stored at −80 °C and 4 °C or after 5 freeze-thaw cycles (Figure 3D). Stability of S protein was further confirmed by DSF data, in which a marginal variation (~1.5 °C) in S protein melting temperatures could be observed between all conditions explored (Figure 3E).

#### 3.2.3. Epitope Mapping of S Protein

The quality of purified S protein was confirmed by epitope mapping. ELISA plates were coated with the protein and epitopes from non-overlapping antigenic clusters on the protein were detected with human monoclonal antibodies known to neutralize SARS-CoV2 with high affinity [31]. The pan-SARS antibody CR3022 [32] and an ACE2-Fc chimeric protein were used to test S protein binding to its receptor (Figure 3F). ELISA data indicate that insect-derived S protein is capable of binding to ACE2 receptor and, importantly, it is recognized by CR3022 monoclonal antibody and by all the other tested anti-S neutralizing antibodies directed toward various epitope clusters.

### 3.3. Conjugation of SARS-CoV-2 Spike Protein to Virosomes

**Figure 4:**
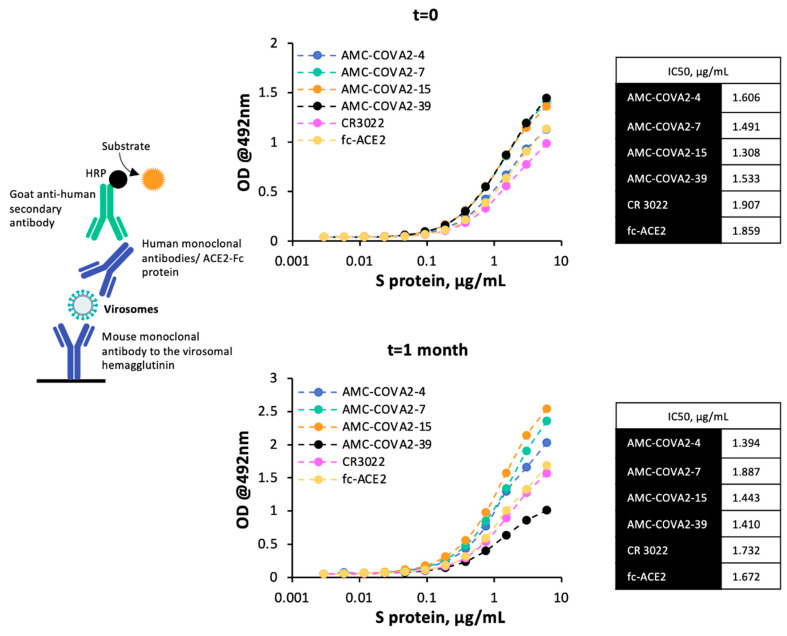
Binding of antibodies to S protein presented on virosomes. Virosomes bound to ELISA plates coated with anti-hemagglutinin were incubated with non-overlapping human neutralizing antibodies recognizing epitopes in the receptor binding domain of S, or with an ACE2-Fc chimera and developed with goat anti-human HRP; panel A at production of virosomes, panel B one month after storage at 4 °C.

Purified S protein was covalently coupled to virosomes through DBCO-azide click chemistry, and the presence of S protein on the virosomes through the exposure of key epitopes on the protein and the binding of an ACE2-Fc were confirmed by ELISA (Figure 4). Results indicate that the S protein as displayed on the surface of the virosomes is capable of binding to the ACE2 receptor and is also recognized by CR3022 and by all the tested neutralizing antibodies toward various epitope clusters. This binding capacity is also preserved on virosomes-S stored at 4 °C for 1 month, as similar IC50 values were obtained for t = 0 and t = 1 month, thus demonstrating an enhanced stability of virosomes-S.

## 4. Discussion

The production of the full-length recombinant S protein of SARS-CoV-2 S has been attempted in mammalian (e.g., HEK293, CHO) and insect (e.g., *Sf*-9 and High Five) cells; however, despite several bioprocess strategies being explored to date e.g., low culture temperature, new cell lines, most induce modest to no improvements in production yields [33,34].

In this study, we have explored different signal peptides, baculovirus transfer vectors, insect cell lines and infection strategies for S protein production. Specific production rates were maximized using BVM signal peptide, pOET5 baculovirus transfer vector, SuperSf9-2 cell line and CCI = 2 × 10^6^ and MOI = 1 pfu/cell. While the cell line is known to enhance expression of highly unstable proteins [20,35], its improved phenotype has never been demonstrated for S protein production; all other variables herein studied have not yet been explored or reported yet to date. Noteworthy is that the production yield achieved was ~4 mg/L, which, to the best of our knowledge, is the highest value obtained for full-length S protein production using IC-BEVS.

In this study, aiming to obtain a stable and folded trimer form of S protein in its pre-fusion conformation, we have combined the elimination of the S1 and S2 furin cleavage site with the addition of double proline mutation to prevent unfolding [36] and the HIV gp160 derived fusion clamp [37]. To further increase S protein stability, particularly upon storage, a screening study of formulation buffers was performed. Buffers B and C, which include glycerol and sucrose as cryoprotectants, respectively, have shown to outperform buffer A (without cryoprotectant) in all conditions tested (i.e., −80 and 4 °C, and after 2 freeze-thaw cycles). Glycerol and sucrose act as stabilizers by inhibiting protein aggregation, shifting the native protein ensemble to more compact states and reducing local backbone fluctuations, resulting in protein stabilization in extreme thermal or chemical environments [38,39]. The list of approved drug products using glycerol as cryoprotectant is extensive as it facilitates drug uptake by the cells while having low toxicity associated [40] and as such, Buffer B (containing glycerol) was selected as the formulation buffer for S protein in our study. S protein thermal stability was assessed by DSF, with melting temperatures varying between 44–46 °C in agreement with literature data [30,41,42]. This method is commonly used to assess protein stability and readily applied for formulation buffer screening [43]. S protein aggregation was assessed using HPLC-SEC, which is one of the more robust and reproducible methods for tracing protein aggregates [44]. The chromatograms showed only one main form of S protein with approximately 400 kDa, consistent with its tertiary structure. Noteworthy, S trimeric form could be maintained for up to 90 days at −80 and 4 °C or after five freeze-thaw cycles, contrasting to other reports in which S protein aggregation was observed after 1 day at 4 °C [30,42].

The results obtained in this study reveal that most N-glycosylation sites in S protein were occupied and that N-glycans included complex/paucimannose glycans and high mannose glycans. In addition, five sites had more than 40% oligomannose type, similar to previous studies using *Sf*-9 cells [15] and mammalian HEK293 cells [17]. The S protein produced in insect cells, either as single antigen or displayed on the surface of the virosomes is bound by ACE2 receptor, pan-SARS antibody CR3022 [32] and neutralizing antibodies to the various epitope clusters [31] in ELISA, as proxy for its antigenicity integrity and biological activity [45]. Additionally, binding capacity was also maintained on virosomes-S stored at 4 °C for 1 month. These results suggest that S protein covalently coupled via its His tag to a click chemistry lipid present in the virosomal membrane results in an oriented display of the protein and properly exposes its receptor binding domain (RBD) involved in ACE2 binding on target cells, thus, theoretically favoring the induction of relevant neutralizing antibodies toward RBD for blocking cell infection.

## 5. Conclusions

This study demonstrates the potential of IC-BEVS for the expression of high-quality SARS-CoV-2 S protein to be included into a virosome-based COVID-19 vaccine candidate. The bioprocessing engineering strategies herein adopted allowed the production of ~4 mg/L of full-length S protein, which, to the best of our knowledge, is the highest value achieved to date using insect cells. In addition, the insect *Sf*-9 cells derived S protein exhibited glycan processing similar to mammalian cells and mid-term stability upon storage. Furthermore, the S protein displayed on the surface of the virosomes was capable of binding to the ACE2 receptor and was recognized by a broad array of neutralizing antibodies, even after storage of the virosomes-S at 4 °C for 1 month. To validate these particles as a COVID-19 vaccine candidate, immunogenicity and safety-toxicology studies in adequate animal models should be performed.

## Figures and Tables

**Table 1:** List of expression plasmids used for baculovirus generation.

Expression Plasmid	Signal Peptide	rBac Transfer Vector
rBac 1	BVM	pOET3
rBac 2	gp67	pOET3
rBac 3	native	pOET3
rBac 4	BVM	pOET1
rBac 5	BVM	pOET5

BVM—honeybee melittin; rBac—recombinant baculovirus.

## Data Availability

The sensitive nature of some of the reagents used in this study (e.g., cell lines, plasmids, baculoviruses, virosomes and antibodies) means that they are only readily available internally to the author’s institutions staff for R&D purposes. For external researchers, approval of reagents request may be obtained via email addressed to the corresponding author.

## Supplementary Materials

The following supporting information can be downloaded at: https://www.mdpi.com/article/10.3390/pharmaceutics14040854/s1, Figure S1: Production of SARS-CoV-2 Spike (S) protein in small-scale shake flasks; Table S1: Glycan types observed across all the N-linked glycosylation sites of SARS-CoV-2 S protein.
