# Supplementary material for: Insect Cells for High-Yield Production of SARS-CoV-2 Spike Protein: Building a Virosome-Based COVID-19 Vaccine Candidate
Source: Pharmaceutics. 2022 Apr 13;14(4):854. doi: 10.3390/pharmaceutics14040854 (PMC9031128; doi:10.3390/pharmaceutics14040854)
Supplement: Supplementary file 1 [file pharmaceutics-14-00854-s001.zip › pharmaceutics-1653903-supplementary.pdf]

Supplementary Information

SARS-CoV-2 Spike (S) protein sequence

MFVFLVLLPLVSSQCVNLTTRTQLPPAYTNSFTRGVYYPDKVFRSSVLHSTQDLFLPFFSNVTWFHA
IHVSGTNGTKRFDNPVLPFNDGVYFASTEKSNIIRGWIFGTTLDSKTQSLLIVNNATNVVIKVCEFQ
FCNDPFLGVYYHKNNKSWMESEFRVYSSANNCTFEYVSQPFLMDLEGKQGNFKNLREFVFKNIDGYF
KIYSKHTPINLVRDLPQGFSALEPLVDLPIGINITRFQTLLALHRSYLTPGDSSSGWTAGAAAYYVG
YLQPRTFLLKYNENGTITDAVDCALDPLSETKCTLKSFTVEKGIYQTSNFRVQPTESIVRFPNITNL
CPFGEVFNATRFASVYAWNRKRISNCVADYSVLYNSASFSTFKCYGVSPTKLNDLCFTNVYADSFVI
RGDEVRQIAPGQTGKIADYNYKLPDDFTGCVIAWNSNNLDSKVGGNYNYLYRLFRKSNLKPFERDIS
TEIYQAGSTPCNGVEGFNCYFPLQSYGFQPTNGVGYQPYRVVVLSFELLHAPATVCGPKKSTNLVKN
KCVNFNFNGLTGTGVLTESNKKFLPFQQFGRDIADTTDAVRDPQTLEILDITPCSFGGVSVITPGTN
TSNQVAVLYQDVNCTEVPVAIHADQLTPTWRVYSTGSNVFQTRAGCLIGAEHVNNSYECDIPIGAGI
CASYQTQTNSPRGSASVASQSIIAYTMSLGAENSVAYSNNSIAIPTNFTISVTTEILPVSMTKTSVD
CTMYICGDSTECSNLLLQYGSFCTQLNRALTGIAVEQDKNTQEVFAQVKQIYKTPPIKDFGGFNFSQ
ILPDPSKPSKRSFIEDLLFNKVTLADAGFIKQYGDCLGDIAARDLICAQKFNGLTVLPPLLTDEMIA
QYTSALLAGTITSGWTFGAGAALQIPFAMQMAYRFNGIGVTQNVLYENQKLIANQFNSAIGKIQDSL
SSTASALGKLQDVVNQNAQALNTLVKQLSSNFGAISSVLNDILSRLDPPEAEVQIDRLITGRLQSLQ
TYVTQQLIRAAEIRASANLAATKMSECVLGQSKRVDFCGKGYHLMSFPQSAPHGVVFLHVTYVPAQE
KNFTTAPAICHDGKAHFPREGVFVSNGTHWFVTQRNFYEPQIITTDNTFVSGNCDVVIGIVNNTVYD
PLQPELDSFKEELDKYFKNHTSPDVDLGDISGINASVVNIQKEIDRLNEVAKNLNESLIDLQELGKY
EQGGSGGIVQQQNNLLRAIEAQQHLLQLTVWGIKQLQARILAGGSGGHTTWMEWDREINNYTSLIHS
LIEESQNQQEKNEQELLEGGSGKHHHHHHCA

Red color – signal peptide native *

Black color – protein

Green color – linker

Blue color- Two sequences corresponding to the N-helix and C-helix of the HIV-1 clade B gp41 ectodomain

Orange color – 6xhistag

Purple color – Furin cleavage site (RRARS) was mutated to (R)GSAS

* other signal peptides used:

MKFLVNVALVFMVVYISFIYA **– bee venom melittin**

MLLVNQSHQGFNKEHTSKMVSAIVLYVLLAAAAHSAFA- **gp67**

Supplementary Figures

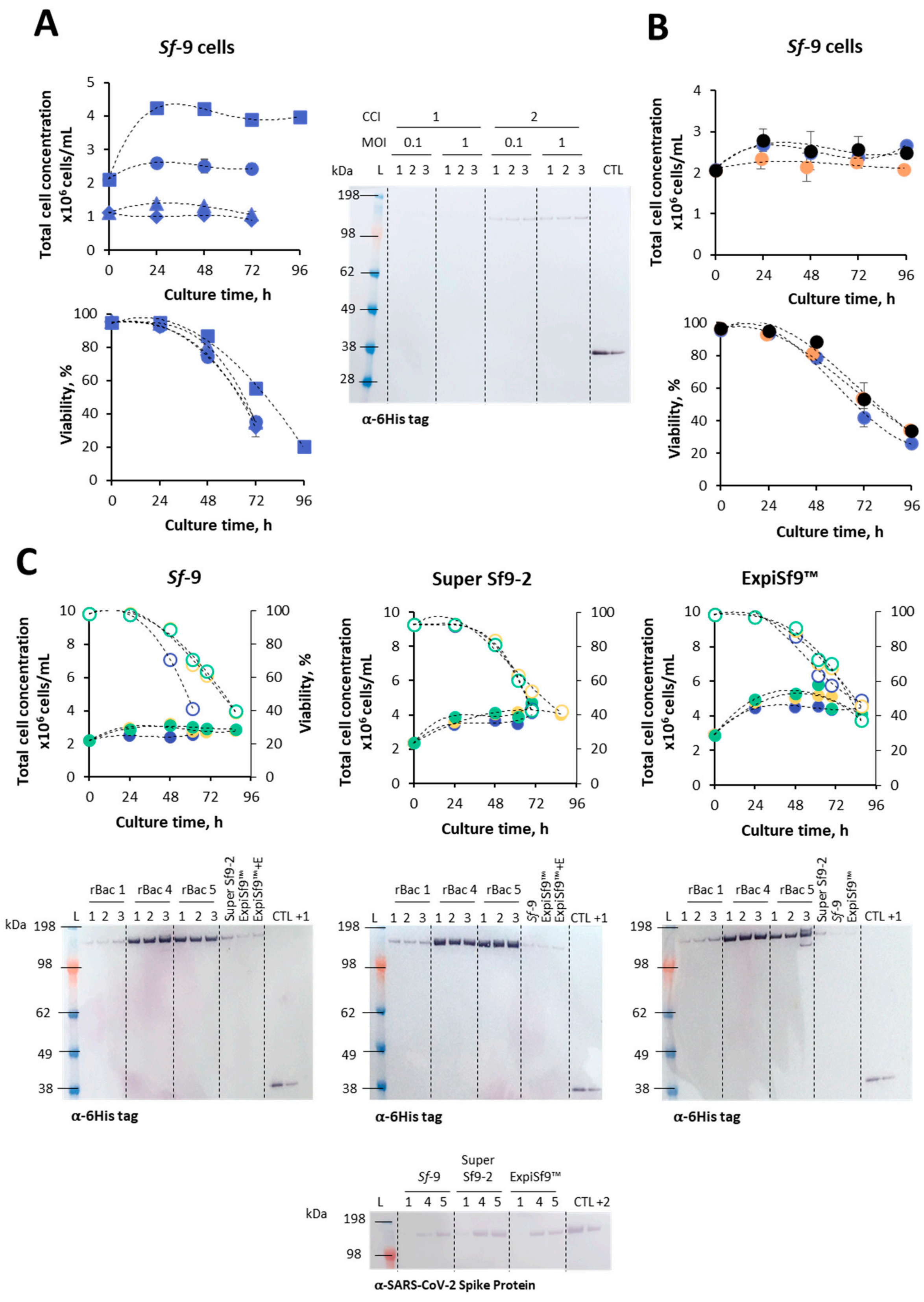

Figure S1. Production of SARS-CoV-2 Spike (S) protein in small-scale shake flasks. (A) Cell growth kinetics and identification of S protein in culture supernatant samples collected at time of harvest by Western blot upon infection with rBac 1 at different MOIs and CCIs; symbol code: CCI=1/MOI=0.1 (triangle), CCI=1/MOI=1 (diamond), CCI=2/MOI=0.1 (square), CCI=2/MOI=1 (circle); (B) Cell growth kinetics upon infection with baculovirus (rBac 1, 2 or 3). Color code: rBac 1 (blue), 2 (orange) or 3 (black). (C) Cell growth kinetics and identification of S protein in culture supernatant samples collected at time of harvest by Western blot upon infection of Sf-9, SuperSf9-2 or ExpiSf9™ cells with rBac 1 (blue), 4 (yellow) or 5 (green). For Western blot analysis, a mouse monoclonal 6-Histag antibody and a human monoclonal SARS-CoV-2 S antibody were used; positive controls were an in-house purified protein with a hexahistidine tag in the C-terminal at 0.2 and 0.1 µg (CTL+1) an in-house purified Spike protein with a hexahistidine tag in the C-terminal at 1 and 0.5 ng (CTL+2); Ladder (L) is SeeBlue™ Plus2 Pre-stained Protein Standard; numbers 1-3 denote culture replicates; numbers 1, 4 and 5 denote rBAC used; the expected MW of S protein monomer is approximately 140 kDa. Data are expressed as mean ± standard deviation (relative to three biological replicates, n=3).

Supplementary Table

Table S1. Glycan types observed across all the N-linked glycosylation sites of SARS-CoV-2 S protein.

Glycosylation Site	Composition	Xic Area	Classification	Compatible structures
N68_N81	HexNAc(2)Hex(2)Fuc(1)	3606883	Complex/paucimannose	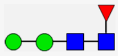
	HexNAc(2)Hex(2)Fuc(2)	127620	Complex/paucimannose	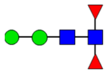
	HexNAc(2)Hex(2)	167684	Complex/paucimannose	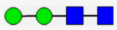
	HexNAc(2)Hex(3)Fuc(1)	3874952	Complex/paucimannose	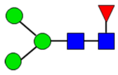
	HexNAc(2)Hex(3)Fuc(2)	127620	Complex/paucimannose	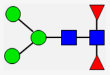
	HexNAc(2)Hex(3)	295304	Complex/paucimannose	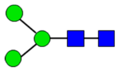
	HexNAc(3)Hex(3)	1232878	Complex/paucimannose	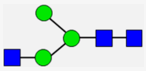
	HexNAc(2)Hex(4)Fuc(1)	3606883	Complex/paucimannose	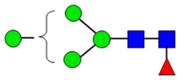
	HexNAc(2)Hex(4)	645825	Complex/paucimannose	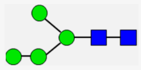
	HexNAc(2)Hex(5)	1360804	High Mannose	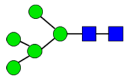
	HexNAc(2)Hex(6)	2524829	High Mannose	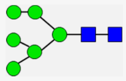
	HexNAc(2)Hex(7)	3294779	High Mannose	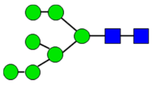
	HexNAc(2)Hex(8)	2399473	High Mannose	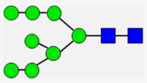
	HexNAc(2)Hex(9)	1096759	High Mannose	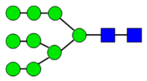
N129	HexNAc(2)Hex(3)	2004097	Complex/paucimannose	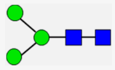

	HexNAc(2)Hex(3)Fuc(1)	2554145	Complex/paucimannose	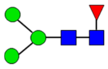
	HexNAc(2)Hex(4)	120010	Complex/paucimannose	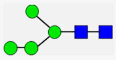
	HexNAc(2)Hex(5)	725195	High Mannose	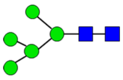
	HexNAc(2)Hex(6)	773796	High Mannose	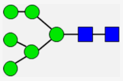
	HexNAc(2)Hex(7)	672100	High Mannose	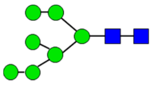
	HexNAc(2)Hex(8)	278065	High Mannose	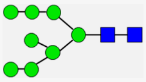
	HexNAc(3)Hex(3)	20115	Complex/paucimannose	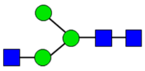
	HexNAc(2)Hex(2)Fuc(1)	65703	Complex/paucimannose	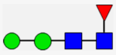
**N156**	HexNAc(2)Hex(3)Fuc(1)	174985	Complex/paucimannose	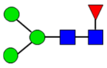
	HexNAc(2)Hex(4)Fuc(1)	43890	Complex/paucimannose	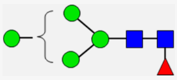
	HexNAc(3)Hex(3)	157110	Complex/paucimannose	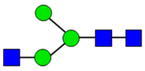
	HexNAc(2)Hex(5)	225936	High Mannose	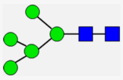
**N172**	HexNAc(2)Hex(3)	134726	Complex/paucimannose	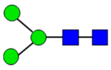
	HexNAc(2)Hex(3)Fuc(1)	142046	Complex/paucimannose	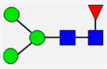
	HexNAc(3)Hex(3)	126546	Complex/paucimannose	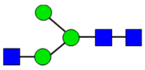
	HexNAc(2)Hex(7)	865017	High Mannose	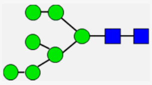
**N241**	HexNAc(2)Hex(3)	109113	Complex/paucimannose	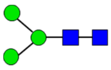
	HexNAc(2)Hex(3)Fuc(1)	352692	Complex/paucimannose	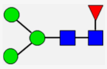
	HexNAc(2)Hex(4)	28186	Complex/paucimannose	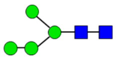

	HexNAc(2)Hex(5)	257580	High Mannose	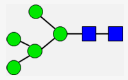
	HexNAc(2)Hex(6)	137358	High Mannose	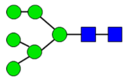
	HexNAc(2)Hex(7)	571035	High Mannose	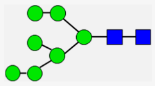
	HexNAc(2)Hex(8)	853685	High Mannose	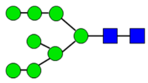
	HexNAc(2)Hex(9)	330840	High Mannose	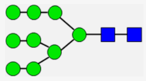
	HexNAc(3)Hex(3)	96245	Complex/paucimannose	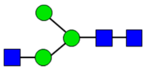
**N289**	HexNAc(2)Hex(2)Fuc(1)	80821	Complex/paucimannose	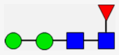
	HexNAc(2)Hex(3)	78663	Complex/paucimannose	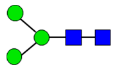
	HexNAc(2)Hex(3)Fuc(1)	2665943	Complex/paucimannose	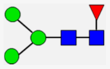
	HexNAc(2)Hex(6)	19052	High Mannose	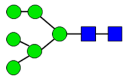
	HexNAc(3)Hex(3)	283902	Complex/paucimannose	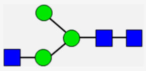
**N338_N350**	HexNAc(2)Hex(2)Fuc(1)	4953764	Complex/paucimannose	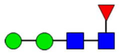
	HexNAc(2)Hex(2)Fuc(2)	3315768	Complex/paucimannose	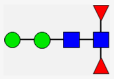
	HexNAc(2)Hex(2)	1606367	Complex/paucimannose	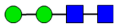
	HexNAc(2)Hex(3)Fuc(1)	11281019	Complex/paucimannose	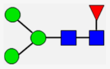
	HexNAc(3)Hex(3)Fuc(1)	284175	Complex/paucimannose	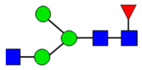
	HexNAc(2)Hex(3)	4363718	Complex/paucimannose	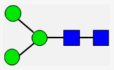
	HexNAc(3)Hex(3)	758097	Complex/paucimannose	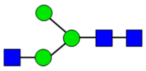
	HexNAc(2)Hex(4)Fuc(1)	4303334	Complex/paucimannose	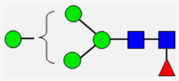

	HexNAc(2)Hex(4)	4508422	Complex/paucimannose	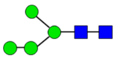
	HexNAc(2)Hex(5)	1607709	High Mannose	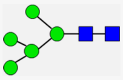
	HexNAc(2)Hex(6)	1702413	High Mannose	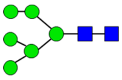
	HexNAc(2)Hex(7)	1253490	High Mannose	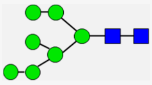
	HexNAc(2)Hex(8)	744393	High Mannose	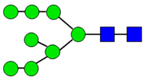
	HexNAc(2)Hex(9)	586592	High Mannose	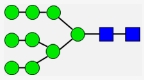
**N610_N623**	HexNAc(2)Hex(2)Fuc(1)	709673	Complex/paucimannose	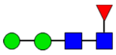
	HexNAc(2)Hex(2)Fuc(2)	709673	Complex/paucimannose	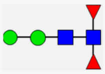
	HexNAc(2)Hex(3)	709673	Complex/paucimannose	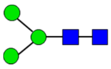
	HexNAc(2)Hex(3)Fuc(1)	1419346	Complex/paucimannose	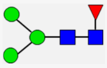
	HexNAc(2)Hex(3)Fuc(2)	709673	Complex/paucimannose	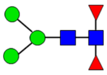
	HexNAc(2)Hex(4)	709673	Complex/paucimannose	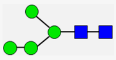
	HexNAc(2)Hex(4)Fuc(1)	709673	Complex/paucimannose	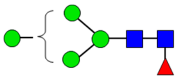
**N664**	HexNAc(2)Hex(3)	462288	Complex/paucimannose	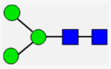
	HexNAc(2)Hex(3)Fuc(1)	1717005	Complex/paucimannose	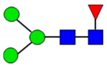
**N808**	HexNAc(2)Hex(2)	4902935	Complex/paucimannose	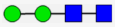
	HexNAc(2)Hex(3)Fuc(1)	1154799	Complex/paucimannose	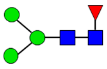
	HexNAc(2)Hex(5)	371956	High Mannose	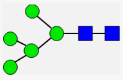
	HexNAc(2)Hex(6)	320235	High Mannose	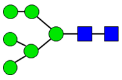

	HexNAc(2)Hex(7)	862139	High Mannose	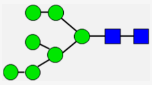
	HexNAc(2)Hex(8)	491635	High Mannose	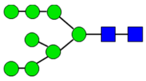
	HexNAc(3)Hex(3)	220173	Complex/paucimannose	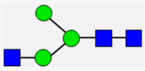
**N1081**	HexNAc(2)Hex(3)	103554	Complex/paucimannose	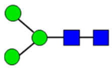
	HexNAc(2)Hex(3)Fuc(1)	378378	Complex/paucimannose	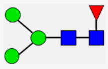
	HexNAc(3)Hex(3)	561390	Complex/paucimannose	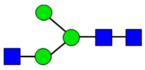
	HexNAc(2)Hex(7)	141765	High Mannose	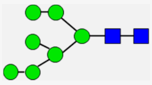
	HexNAc(2)Hex(8)	152912	High Mannose	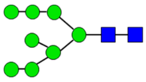
**N1105**	HexNAc(2)Hex(3)	6579775	Complex/paucimannose	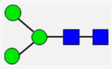
	HexNAc(2)Hex(3)Fuc(1)	1110246	Complex/paucimannose	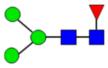
	HexNAc(2)Hex(5)	217883	High Mannose	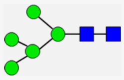
	HexNAc(2)Hex(6)	76321	High Mannose	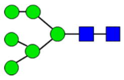
	HexNAc(2)Hex(7)	85324	High Mannose	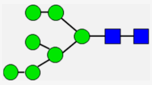
	HexNAc(2)Hex(8)	69084	High Mannose	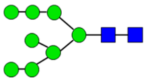
	HexNAc(3)Hex(3)	89839	Complex/paucimannose	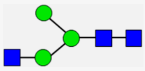
	HexNAc(2)Hex(2)	439181	Complex/paucimannose	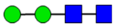
**N1141**	HexNAc(2)Hex(3)Fuc(1)	104410	Complex/paucimannose	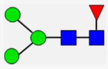
**N1165_1180**	HexNAc(2)Hex(3)	1053129	Complex/paucimannose	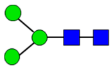
	HexNAc(2)Hex(2)Fuc(1)	4134153	Complex/paucimannose	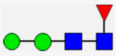

	HexNAc(2)Hex(4)	1059599	Complex/paucimannose	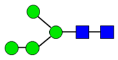
	HexNAc(2)Hex(4)Fuc(1)	1005983	Complex/paucimannose	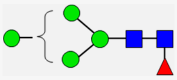
	HexNAc(2)Hex(7)	31537	High Mannose	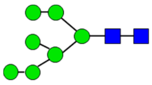
	HexNAc(2)Hex(8)	47146	High Mannose	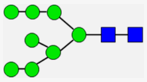
	HexNAc(3)Hex(3)	47146	Complex/paucimannose	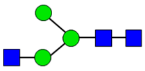
	HexNAc(3)Hex(3)Fuc(2)	97567	Complex/paucimannose	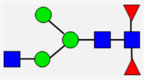
	HexNAc(2)Hex(2)Fuc(2)	1005983	Complex/paucimannose	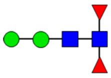
**N1201**	HexNAc(3)Hex(3)Fuc(2)	561417	Complex/paucimannose	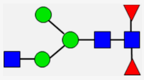
	HexNAc(2)Hex(3)	522918	Complex/paucimannose	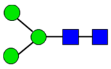
	HexNAc(2)Hex(3)Fuc(1)	5146552	Complex/paucimannose	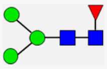
	HexNAc(2)Hex(3)Fuc(2)	35771	Complex/paucimannose	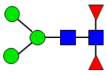
	HexNAc(2)Hex(4)	41774	Complex/paucimannose	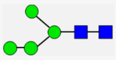
	HexNAc(2)Hex(4)Fuc(1)	44295	Complex/paucimannose	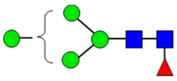
	HexNAc(2)Hex(5)	49439	High Mannose	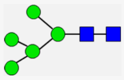
	HexNAc(2)Hex(6)	63021	High Mannose	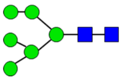
	HexNAc(2)Hex(7)	96451	High Mannose	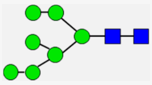
	HexNAc(2)Hex(8)	37523	High Mannose	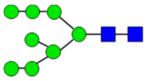
	HexNAc(3)Hex(3)	848426	Complex/paucimannose	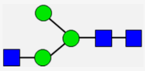

	HexNAc(2)Hex(2)Fuc(1)	345388	Complex/paucimannose	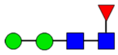
**N1294**	HexNAc(2)Hex(3)Fuc(1)	71142	Complex/paucimannose	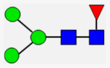
